# Detoxification of Mitochondrial Oxidants and Apoptotic Signaling Are Facilitated by Thioredoxin-2 and Peroxiredoxin-3 during Hyperoxic Injury

**DOI:** 10.1371/journal.pone.0168777

**Published:** 2017-01-03

**Authors:** Benjamin J. Forred, Darwin R. Daugaard, Brianna K. Titus, Ryan R. Wood, Miranda J. Floen, Michelle L. Booze, Peter F. Vitiello

**Affiliations:** 1 Children’s Health Research Center, Sanford Research, Sioux Falls, South Dakota, United States of America; 2 Department of Pediatrics, University of South Dakota Sanford School of Medicine, Sioux Falls, South Dakota, United States of America; University of Nebraska-Lincoln, UNITED STATES

## Abstract

Mitochondria play a fundamental role in the regulation of cell death during accumulation of oxidants. High concentrations of atmospheric oxygen (hyperoxia), used clinically to treat tissue hypoxia in premature newborns, is known to elicit oxidative stress and mitochondrial injury to pulmonary epithelial cells. A consequence of oxidative stress in mitochondria is the accumulation of peroxides which are detoxified by the dedicated mitochondrial thioredoxin system. This system is comprised of the oxidoreductase activities of peroxiredoxin-3 (Prx3), thioredoxin-2 (Trx2), and thioredoxin reductase-2 (TrxR2). The goal of this study was to understand the role of the mitochondrial thioredoxin system and mitochondrial injuries during hyperoxic exposure. Flow analysis of the redox-sensitive, mitochondrial-specific fluorophore, MitoSOX, indicated increased levels of mitochondrial oxidant formation in human adenocarcinoma cells cultured in 95% oxygen. Increased expression of Trx2 and TrxR2 in response to hyperoxia were not attributable to changes in mitochondrial mass, suggesting that hyperoxic upregulation of mitochondrial thioredoxins prevents accumulation of oxidized Prx3. Mitochondrial oxidoreductase activities were modulated through pharmacological inhibition of TrxR2 with auranofin and genetically through shRNA knockdown of Trx2 and Prx3. Diminished Trx2 and Prx3 expression was associated with accumulation of mitochondrial superoxide; however, only shRNA knockdown of Trx2 increased susceptibility to hyperoxic cell death and increased phosphorylation of apoptosis signal-regulating kinase-1 (ASK1). In conclusion, the mitochondrial thioredoxin system regulates hyperoxic-mediated death of pulmonary epithelial cells through detoxification of oxidants and regulation of redox-dependent apoptotic signaling.

## Introduction

Transitioning from an *in utero* environment to life outside the womb is marked by change from a relatively hypoxic environment to an oxygen-rich atmosphere. Lungs of prematurely born infants are underdeveloped with fewer alveoli and lower expression of antioxidant enzymes [1]. Consequently, preterm infants are at a disadvantage in coping with this oxidative transition, even before therapeutic interventions such as supplemental oxygen (hyperoxia) and mechanical ventilation are considered. Bronchopulmonary dysplasia (BPD) is caused, in part, by sustained oxygen therapy in preterm infants and is marked by alveolar simplification [2, 3]. Disrupted perinatal alveolar growth may be mediated by loss of alveolar type 2 (AT2) epithelial cells through either apoptosis or altered programming associated with exposure to or recovery from excess oxygen [4–6]. Hyperoxic cellular injuries are manifested in part through generation and accumulation of reactive oxygen species (ROS) [7]. Although BPD is associated with prematurity, ROS generation and oxidative injury to the alveolar epithelium is also a component of additional lung diseases such as acute respiratory distress syndrome and acute lung injury [8].

Mitochondria are considered a major site of ROS production since ρ^0^ cells fail to generate ROS during hyperoxic culture [9, 10]. Electron transport chain complexes I and III are likely the predominant sources of mitochondrial ROS production during hyperoxic injury [11]. Although ROS accumulation may not be an absolute determinant, oxidants are thought to promote hyperoxic-mediated cell death through activation of pro-apoptotic Bcl-2 family proteins [12]. Cells deficient in *Bax* or *Bak* do not undergo apoptosis in response to hyperoxia [10, 13]. Furthermore, anti-apoptotic Bcl-2 proteins such as Bcl-X_L_ and Mcl-1 can abrogate Bax-dependent hyperoxic cell death [14–16]. Collectively, these data support a functional relationship between respiring mitochondria and activation of pro-apoptotic Bcl-2 proteins which may mediate susceptibility to hyperoxic cell death.

Multiple endogenous enzyme systems work in coordination based on both target specificity and catalytic rate to maintain redox homeostasis in the mitochondrial matrix. Superoxide radical formed as a byproduct of oxidative phosphorylation (OXPHOS) is rapidly converted by superoxide dismutase 2 (SOD2) to hydrogen peroxide. Peroxiredoxin-3 (Prx3) is the primary oxidoreductase for reducing hydrogen peroxide in the matrix and reacts more efficiently than either catalase or glutathione peroxidase. In fact, it is estimated that Prx3 scavenges 90% of all peroxides in the mitochondrial matrix [17]. However, Prx3 can also reduce peroxynitrite, alkylhydroperoxides, as well as peroxides found on amino acids and proteins [18]. Similar to all 2-cysteine peroxiredoxins, the peroxidatic cysteine of Prx3 reacts with hydrogen peroxide to first form a sulfenic acid intermediate. This subsequently forms a disulfide with a resolving cysteine to form the oxidized Prx3 homodimer [19]. Regeneration of reduced monomeric Prx3 is dependent on thioredoxin-2 (Trx2), an enzyme which is recycled by thioredoxin reductase-2 (TrxR2) using electrons from NADPH. Because of its fundamental role in peroxide detoxification, the oxidoreductase activity of Trx2 prevents mitochondrial-dependent cell death mediated by various oxidant stimuli, including tert-butylhydroperoxide, etopiside, kanamycin, TNF-α, and excitotoxicity [20–25]. One proposed mechanism of cytoprotection against oxidative damage is that Trx2 binds and inhibits apoptosis signal-regulating kinase-1 (ASK1) [26, 27]. Trx2 oxidation or displacement by thioredoxin interacting protein (TXNIP) relieves ASK1 inhibition which subsequently initiates caspase-dependent apoptosis [28]. Alternatively, Trx2 may directly prevent activation of the mitochondrial permeability transition pore (MPTP) [23].

In this study we examined the role of Trx2 and Prx3 during hyperoxic injury in human lung epithelial cells. Oxidized Trx2 and Prx3 accumulated during hyperoxia which was associated with increased mitochondrial ROS (mtROS). Furthermore, genetic or pharmacologic inhibition of redox cycling via TrxR2 increased susceptibility to hyperoxic cell death and was associated with generation of mtROS and activation of the MPTP. Only Trx2 disruption, and not Prx3, correlated with activation of pro-apoptotic signaling via ASK1. Our data indicate that Prx3 serves as a primary sensor of mtROS to relieve Trx2 inhibition of ASK1, thereby promoting hyperoxic cell death.

## Materials and Methods

### Cell Culture and Treatment

Human lung adenocarcinoma A549 [29] and H1299 [30] cells (obtained in October 2009 from ATCC; CCL-185 and CRL-5803) were cultured in 5% CO_2_ at 37°C in high glucose (25 mM) DMEM with 10% fetal bovine serum, 50 U/mL penicillin, 50 μg/mL streptomycin and 20 μg/mL gentamycin. During hyperoxic exposure, cells were cultured in a Plexiglas chamber flooded with 95% O_2_/5% CO_2_. For treatments, cells were pre-treated with indicated doses of MitoTEMPO and auranofin (AFN; Sigma Aldrich) prior to hyperoxic exposure. During chronic exposures, MitoTEMPO was supplemented every 24 hrs without changing culture media.

### Flow Cytometry

Cells were pulsed with 5 μM MitoSOX Red or 1 nM TMRE (tetramethylrhodamine, ethyl ester) and 10 nM TO-PRO-3 (Thermo Fisher Scientific). A minimum of 10,000 live events were collected using an Accuri C6 (BD Biosciences) and analyzed using CSampler (BD Biosciences) and FCS Express4 Flow Research edition software (DeNovo). For MitoSOX Red and TMRE experiments, dyes were excited at 488 nm and emitted fluorescence was detected using the FL2 (585/40 nm) and the FL3 (670 LP) filters, respectively.

### SDS-PAGE & Immunoblot

As previously described [31], cell lysates were diluted in Laemmli buffer, separated by polyacrylamide gel electrophoresis (SDS-PAGE), and transferred to PVDF membranes. Membranes were blocked in 5% non-fat dry milk before incubating overnight at 4˚C in rabbit polyclonal anti-Trx2 (1:250, Santa Cruz, sc-50336), rabbit polyclonal anti-Prx3 (1:1,000, Thermo Fisher Scientific, LF-PA0030), rabbit polyclonal anti-TrxR2 (1:1,000, Abcam, ab58445), rabbit polyclonal anti-ASK1 (1:500, Santa Cruz, sc-7931), rabbit polyclonal anti-pASK1 (1:1,000, Cell Signaling, 3765), rabbit polyclonal anti-Bax (1:1,000, Santa Cruz, sc-493), mouse monoclonal anti-Bak (1:1,000, EMD Millipore, AM03) or rabbit polyclonal anti-β-actin (1:1,000, Sigma Aldrich, A2066). Membranes were then incubated with anti-rabbit or anti-mouse (1:5,000, Southern Biotechnology) HRP-conjugated secondary antibodies and immune complexes were detected by chemiluminescence captured and analyzed on a UVP bioimaging system (Upland, CA). Semi-quantitative analyses of immunoblots were performed using Image Studio (LI-COR).

### Quantitative Real-Time PCR (qPCR)

RNA was isolated using the SV Total RNA Isolation System (Promega). cDNA was synthesized from samples with an RNA integrity number of 8.0 or greater evaluated with a 2100 Bioanalyzer (Agilent Technologies). Primers and probes for qPCR were designed for human Prx3, Trx2, TrxR2 and glyceraldehyde-3-phosphate dehydrogenase (GAPDH) using Beacon Designer 7.91 (Premier Biosoftware) (S1 Table). As previously described [31], primers (900 nM) and probes (250 nM) were diluted in 2X Absolute Blue master mix (Thermo Fisher Scientific) and assayed using a 7500 Real Time PCR System (ABI). Standard MIQE guidelines including internal primer validation through mass normalization, assessment of genomic DNA contamination, and assay efficiency were performed [32].

### Mitochondrial Mass

To quantify variation in mitochondrial number as cells are cultured in hyperoxia, DNA was isolated from A549 over a four day hyperoxic timecourse. Phenol/chloroform isolated DNA was analyzed by real-time PCR using commercially available probes designed against the D-loop region of mitochondrial DNA and the *COX1* mitochondrial gene (Fisher Scientific). Input template was mass normalized and mitochondrial Ct values were normalized to the corresponding Ct values for *β-2-microglobulin* (β2M), a nuclear DNA target. Primers and probes for β2M were designed to only amplify genomic DNA (S2 Table).

### Redox Immunoblots

For Trx2 redox status, cells were lysed in 10% ice-cold trichloroacetic acid, washed in acetone, and protein pellets were resuspended in 20 mM Tris-HCl (pH 8.0) containing 15 mM AMS (4-acetoamido-4′-maleimidylstilbene-2,2′-disulphonic acid). Following AMS labeling, lysates were diluted in non-reducing Laemmli buffer and subjected to SDS-PAGE/immunoblot [33]. Trx2 redox potential (E_h_) was determined using the Nernst equation with E0 = -330 mV for Trx2 at pH 7.6 and 25°C [34]. For Prx3 analysis, cells were lysed in 100 mM N-ethylmaleimide (NEM) alkylation buffer supplemented with 10 μg/mL catalase to label and retain reduced monomers for non-reducing SDS-PAGE/immunoblot analysis [35].

### Thioredoxin Reductase Activity Assay

For TrxR2 activity, a commercially available insulin reduction assay was utilized (Cayman Chemical). Cells were harvested in assay buffer (0.1 M NaPO_4_, 5 mM EDTA, 0.1% Triton X-100, 1% protease inhibitor cocktail (Sigma Aldrich), 1% phosphatase inhibitor cocktails 2 and 3 (Sigma Aldrich), and 0.1 mM PMSF) and rotated for 2 hours at 4°C. Lysates were then spun at 10,000 rpm for 15 minutes at 4°C. Supernatants were collected and protein concentration was determined by BCA. Reductase activity was measured in a 96-well plate according to manufacturer’s instructions. Absorbance was measured at 412nm using a Spectramax M5 microplate reader.

### ShRNA Lentiviral Delivery

ShRNA sequences targeting human Trx2 and Prx3 as well as a non-targeting control were designed using BLOCK-iT RNAi Designer software from Thermo Fisher Scientific (S3 Table) and were cloned into the pLVX-shRNA2 (Clontech, Mountain View, CA). Following the manufacturer’s instructions for the Lenti-X system, Lenti-X 293T cells were transfected with pLVX-shRNA2 and HTX packaging mix to produce ≥2.5x10^8^ IFU/mL as determined by qPCR. Cells were seeded in 6-well dishes and transduced with 0.5mL viral media supplemented with 8 μg/mL polybrene for 6 hours. Transduction efficiency after 48 hours was ≥85% by flow cytometry for ZsGreen1.

### Statistical Analyses

Values represent mean ± standard deviation of biological replicates. Group means were compared by 1-way ANOVA using Bonferroni’s post hoc test with GraphPad Prism 5 (GraphPad Software). Statistical significance was defined as P≤0.05.

## Results

### Generation of Mitochondrial Oxidants during Hyperoxic Injury

**Fig 1:**
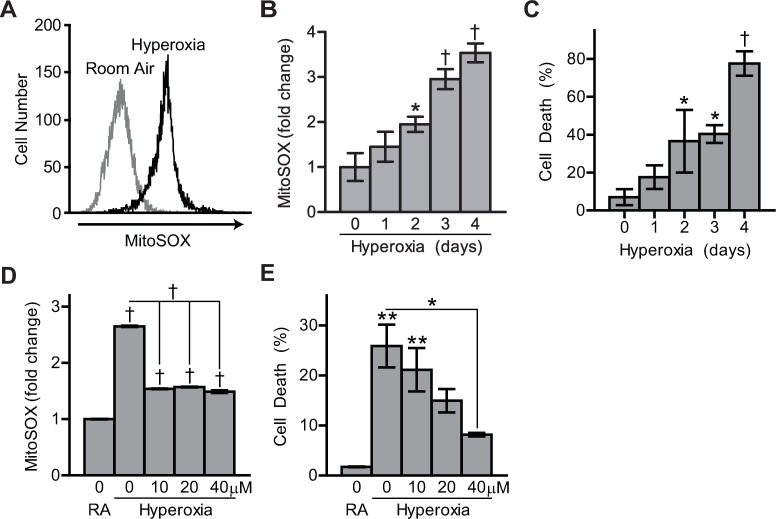
Mitochondrial oxidants promote hyperoxic cell death. MitoSOX was used to quantify mitochondrial oxidants in A549 cells cultured in hyperoxia. (A) MitoSOX red fluorescence intensity and (B) fold-change after 2 days of hyperoxic culture and during a hyperoxic time course. (C) Hyperoxic cell death measured via flow cytometry using TOPRO-3. (D) Fold-change in MitoSOX red fluorescence intensity of A549 cells after 2 days of hyperoxic culture with increased concentrations of MitoTEMPO supplemented in the media. (E) Death of A549 cells cultured concurrently with 3 days hyperoxia and MitoTEMPO supplementation. Data are expressed as mean ± standard deviation of 3 biological replicates analyzed by one-way ANOVA. Statistical significance was defined as *p<0.05, **p<0.01, and †p<0.001.

MitoSOX was used as an indicator of mitochondrial oxidant generation during hyperoxic culture [36, 37]. Time-dependent increases in MitoSOX signal intensity were detected in A549 lung epithelial cells cultured in 95% oxygen (Fig 1A and 1B). Cell death following hyperoxia was quantified using the cell-impermeable fluorescent dye, TO-PRO-3. TO-PRO-3 positive cells were considered dead while debris was excluded from analysis (S1 Fig). Time-dependent increases in cell death were observed over four days of hyperoxia which culminated in an 11-fold increase in cell death after four days (Fig 1C). To determine if detoxification of mitochondrial superoxide alleviated hyperoxic cell death, additional viability experiments were conducted during supplementation with the mitochondrial-targeted superoxide scavenger, MitoTEMPO. Cells simultaneously treated MitoTEMPO and hyperoxia had significantly reduced MitoSOX signal intensity and decreased cell death (Fig 1D and 1E). This data suggests a crucial role for mitochondrial oxidation in hyperoxic cell death.

### Hyperoxic Modulation of Trx2 and Prx3 Expression and Activity

**Fig 2:**
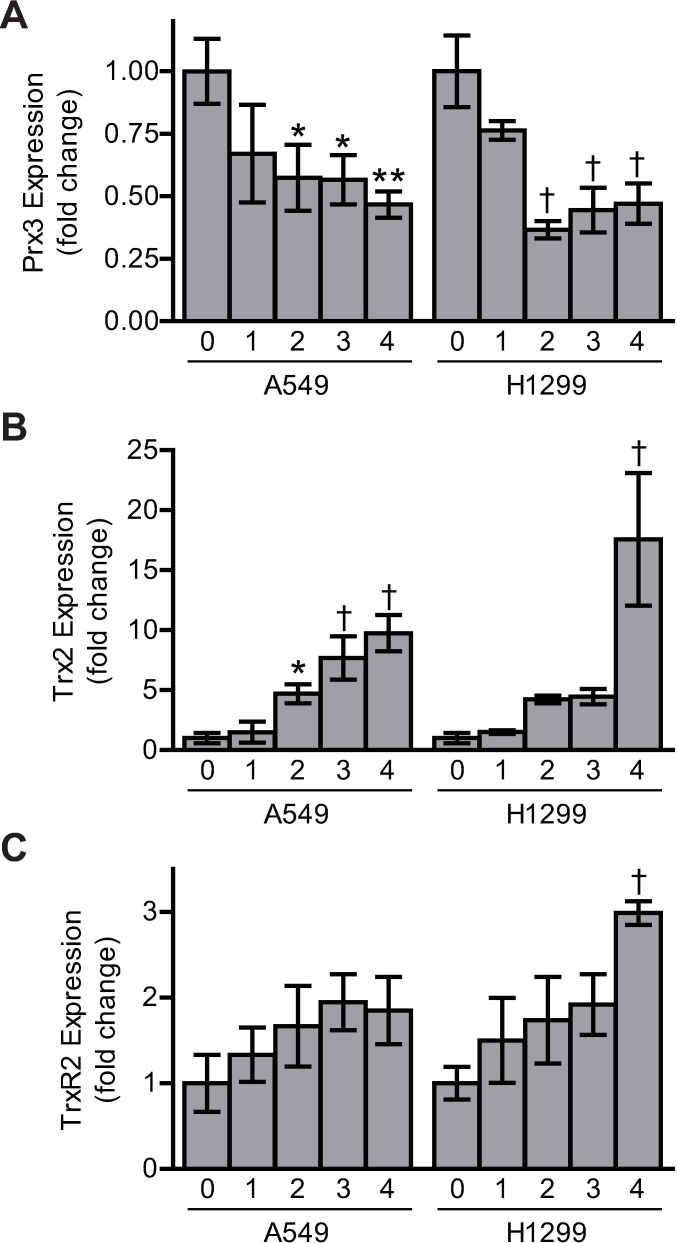
Oxygen-dependent gene expression of Prx3, Trx2, and TrxR2. qPCR analysis of (A) Prx3, (B) Trx2, and (C) TrxR2 expression in A549 and H1299 cells during hyperoxic culture using GAPDH as a loading control. Data are expressed as mean ± standard deviation of 3–4 biological replicates analyzed by one-way ANOVA. Statistical significance was defined as *p<0.05, **p<0.01, and †p<0.001.

**Fig 3:**
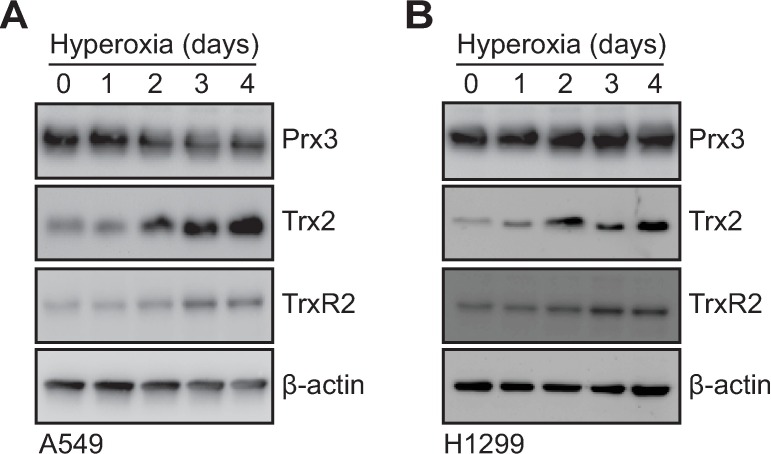
Protein expression of mitochondrial redoxins Prx3, Trx2, and TrxR2 during hyperoxia. Representative SDS-PAGE/immunoblots of cell lysates probed for Prx3, Trx2, and TrxR2 with β-actin as a loading control in (A) A549 and (B) H1299 human lung adenocarcinoma cell lines cultured for increasing days in hyperoxia. Images are representative of 3 independent biological replicates.

Hydrogen peroxide accumulation results from dismutation of superoxide by SOD2 in the mitochondrial matrix [38]. Therefore, we hypothesized that the expression and redox state of Prx3, Trx2, and TrxR2 may be altered as these redox enzymes function as a system to scavenge mitochondrial hydrogen peroxide. Transcript and protein expression of these genes were examined in two human lung epithelial cell lines, A549 and H1299. Although Prx3 gene expression decreased by approximately 50% in both cell lines, surprisingly, Trx2 expression increased nearly 10-fold in hyperoxia (Fig 2A and 2B). This coincided with a significant, albeit smaller, increase in TrxR2 expression (Fig 2C). Similar changes in Prx3, Trx2, and TrxR2 protein expression in both cell lines were detected by immunoblot (Fig 3A and 3B) and quantified (S2 Fig).

**Fig 4:**
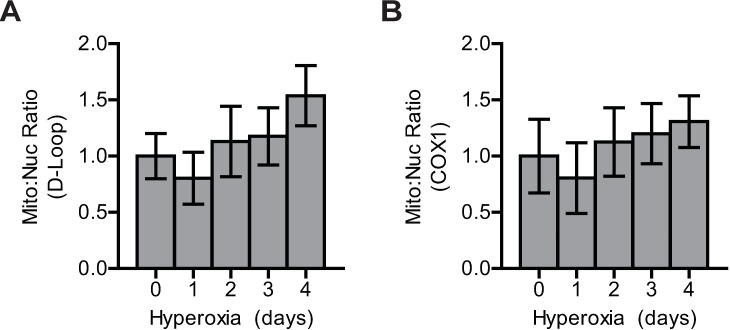
Hyperoxia does not alter mitochondrial mass. Ratio of mitochondrial:nuclear Ct values for (A) *D-Loop* and (B) *COX1* after normalization to *β2M* quantified by qPCR in A549 during hyperoxic culture. Data are expressed as mean ± standard deviation of 3 biological replicates analyzed by one-way ANOVA.

One simple explanation for increased MitoSOX red fluorescence intensity and upregulation of Trx2 and TrxR2 could be attributed to increased mitochondrial mass in response to extended hyperoxic culture [39]. To investigate this possibility, mitochondrial and nuclear DNA were isolated from A549 cells over four days of culture in hyperoxia. DNA was analyzed by real-time PCR using probes designed against two regions of mitochondrial DNA (mtDNA): *cytochrome C oxidase 1* (COX1) and the control region (D-Loop) [40]. A primer and probe set were designed to amplify genomic DNA of the *β-2-microglobulin* (β2M) gene. MtDNA Ct values were normalized to corresponding *β2M* Ct values to derive mitochondrial:nuclear (mito:nuc) ratios for each sample. As a control experiment validating this method, treatment with dideoxycytidine or ethidium bromide expectedly decreased mito:nuc ratio determined by amplifying either D-Loop or *COX1* (S3 Fig). No significant changes in mito:nuc ratio for either of the two mitochondrial genes were observed over the course of the hyperoxic exposure (Fig 4A and 4B). These data suggest that hyperoxic upregulation of mitochondrial thioredoxins and increased MitoSOX red fluorescence signal intensity are the consequence of a cellular response to increased production of mitochondrial oxidants and are not due to changes in mitochondrial number.

**Fig 5:**
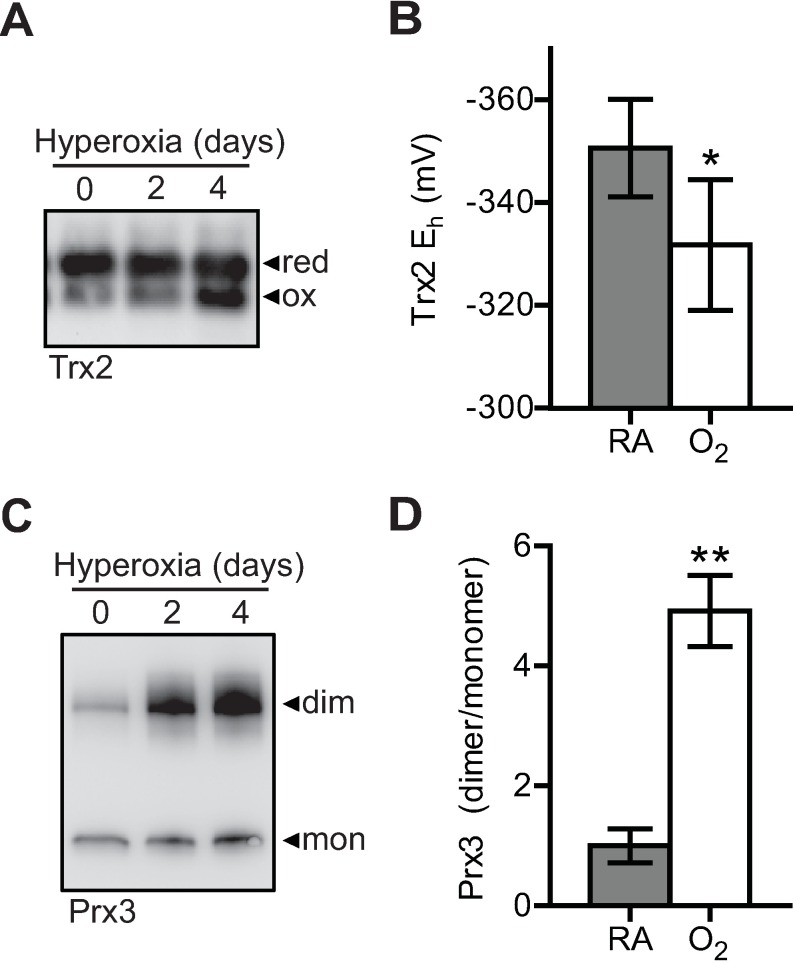
Trx2 and Prx3 oxidation during hyperoxia. (A,C) Differential Trx2 and Prx3 thiol labeling by AMS and NEM respectively, and (B,D) representative detection by SDS-PAGE/immunoblot in A549 cells after treatment with hyperoxia. Images are representative of 3–4 independent biological replicates.

The oxidation state of the catalytic cysteines in Prx3 and Trx2 was monitored by differential thiol alkylation followed by SDS-PAGE. For Trx2 detection, free thiols were labeled with AMS, which causes a mass shift in the reduced form (S4A Fig) [22, 34]. As expected, Trx2 was predominantly reduced under control conditions and became oxidized after 4 days of hyperoxic culture (Fig 5A). Using the Nernst equation [34], Trx2 redox potential (E_h_) was significantly decreased during hyperoxia (Fig 5B). Oxidized Prx3 dimers were stabilized by alkylation of free thiols with NEM, and detection of Prx3 monomers and dimers under non-reducing conditions (S4B Fig) [35]. Prx3 oxidation is detected after 2 days of hyperoxia, which can be expected based on the very fast kinetic rate for the neutralization of hydrogen peroxide by peroxiredoxins (Fig 5C and 5D) [17, 41]. It is important to note that these data do not conflict with Prx3 expression as anti-peroxiredoxin antibodies are thought to have differential reactivity with oxidized and reduced forms [35].

### Trx2 Prevents Hyperoxia-Induced Apoptotic Signaling

**Fig 6:**
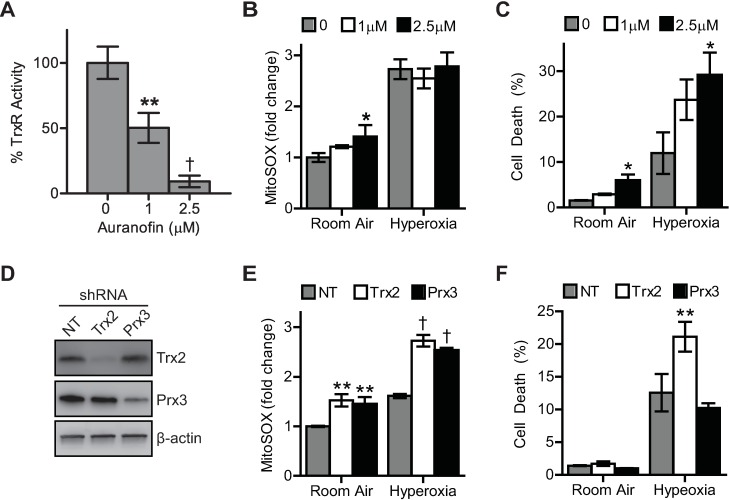
Trx2 inhibition sensitizes cells to hyperoxic cell death. (A) Inhibition of TrxR activity in A549 cells cultured for 24 hours with 1 or 2.5 μM AFN. (B) Fold-change in MitoSOX red fluorescence intensity after 2 days hyperoxic culture and (C) TO-PRO-3 labeling after 3 days of hyperoxic culture with 1 or 2.5 μM AFN. (D) SDS-PAGE/immunoblot of A549 cell lysates for Trx2 and Prx3 protein expression 2 days following lentiviral delivery of non-targeting (NT) and Trx2- or Prx3-targeting shRNAs. (E) Fold-change MitoSOX red fluorescence intensity after 2 days of hyperoxia and (F) TO-PRO-3 labeling after 3 days hyperoxic culture following lentiviral transduction of NT, Trx2, or Prx3 shRNAs in A549 cells. Data are expressed as mean ± standard deviation of 3 biological replicates analyzed by one-way ANOVA. Statistical significance was defined as *p<0.05, **p<0.01, and †p<0.001 (n = 3).

Pharmacologic and genetic approaches were used to determine if inhibition of Trx2 and/or Prx3 activity augmented hyperoxic mitochondrial injuries. Auranofin (AFN), a thioredoxin reductase inhibitor, is known to enter mitochondria, causing Prx3 oxidation (presumably via inhibition of Trx2 redox cycling) and MPTP activation [35, 42, 43]. As expected, AFN exhibited dose-dependent TrxR inhibition in A549 cells (Fig 6A). Culture in 2.5 μM AFN inhibited 91% of TrxR activity. Control cells cultured with AFN in room air had significantly increased MitoSOX red fluorescence intensity. Hyperoxic treatment resulted in more robust fluorescent signal which was not further exacerbated by AFN (Fig 6B). However, AFN supplementation augmented hyperoxic-dependent cell death (Fig 6C). AFN does not have specificity for TrxR2 alone; rather, it inhibits TrxR1 as well. As such, AFN-mediated cell death in the absence of increased MitoSOX red fluorescence may indeed be explained by TrxR1, and not TrxR2, inhibition. Since cytosolic Trx1 is known to sensitize cells to hyperoxic damage [31], a second set of experiments was conducted to genetically target Trx2 and Prx3 via RNAi knockdown. Cells were transduced with lentivirus expressing a non-targeting control or Trx2 and Prx3 targeting shRNAs. ShRNA delivery resulted in knockdown of the specific target without altering expression of the other key mitochondrial redox enzymes (Fig 6D). Knockdown of either Trx2 or Prx3 caused increased MitoSOX red fluorescence intensity in control cells and had a similar effect during hyperoxic culture (Fig 6E). Only knockdown of Trx2 caused increased hyperoxic-induced cell death (Fig 6F).

**Fig 7:**
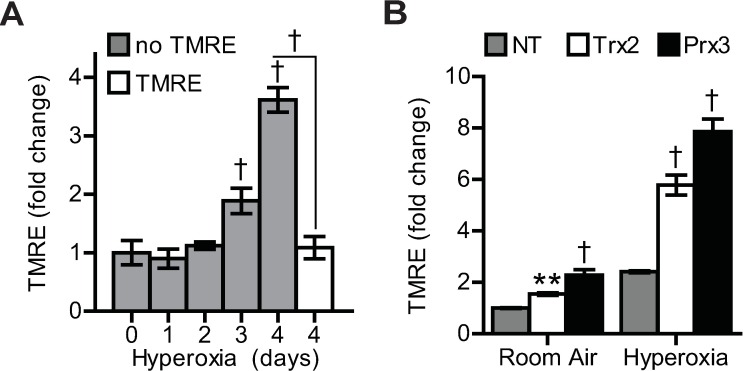
Hyperoxia-induced mitochondrial hyperpolarization. (A) Fold-change in TMRE fluorescence intensity of A549 cells cultured in hyperoxia in the absence or presence of 0.5 μM FCCP. (B) TMRE fluorescence intensity after 3 days hyperoxic culture following lentiviral transduction of NT, Trx2, or Prx3 shRNAs in A549 cells. Data are expressed as mean ± standard deviation of 3 biological replicates analyzed by one-way ANOVA. Statistical significance was defined as **p<0.01, and †p<0.001.

Based on the results of our viability experiments with Trx2 shRNA treatment during hyperoxia, we hypothesized that Trx2 knockdown may cause MPTP activation [23] and used TMRE as an indicator of mitochondrial membrane potential (Δψ_m_). Hyperoxic culture resulted in mitochondrial hyperpolarization (as opposed to depolarization and subsequent MPTP opening), indicated by increased TMRE signal intensity (Fig 7A). To demonstrate specificity, TMRE signal was ablated after incubation for 15 minutes with the proton ionophore, FCCP (carbonyl cyanide 4-(trifluoromethoxy) phenylhydrazone), following hyperoxic culture (Fig 7A) [44]. However, mitochondrial hyperpolarization is associated with mitochondrial hypertrophy, a hallmark of hyperoxic injury [45]. Knockdown of both Trx2 and Prx3 caused increased mitochondrial hyperpolarization in A549 cells cultured in room air and hyperoxia (Fig 7B).

**Fig 8:**
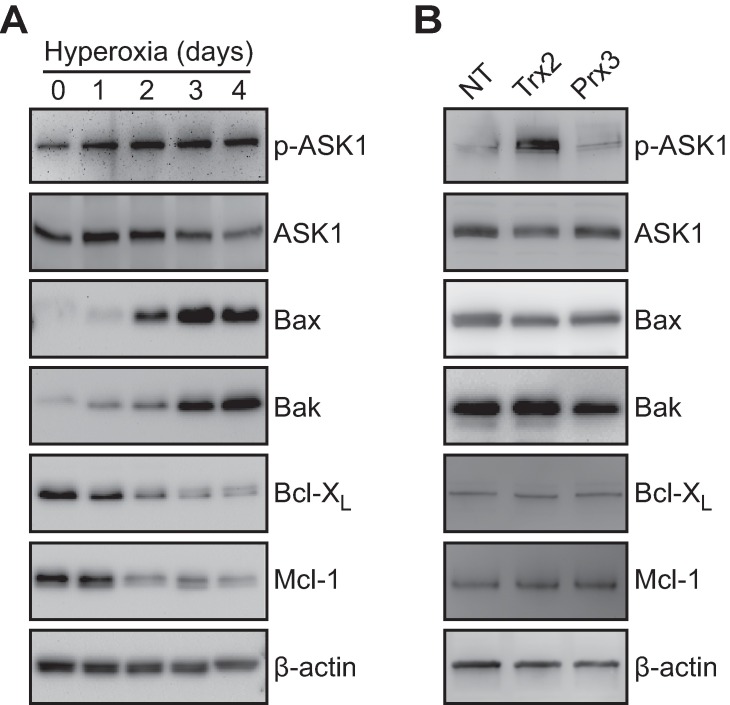
Trx2 inhibits hyperoxic phosphorylation of ASK1. Expression of pro-apoptotic proteins by SDS-PAGE/immunoblot in A549 cells during (A) hyperoxic culture. (B) Immunoblots of lysates from A549 cultured in hyperoxia for two days following lentiviral delivery of NT, Trx2, or Prx3 shRNAs. Images are representative of 3 independent biological replicates.

We also hypothesized that loss of Trx2 may cause susceptibility through loss of ASK1 inhibition [26, 27, 46]. Cells cultured in hyperoxia had increased ASK1 phosphorylation at Thr845, which coincided with increased expression of pro-apopototic Bax and Bak and reduced expression of anti-apoptotic Bcl-X_L_ and Mcl-1 (Fig 8A). Knockdown of Trx2 in hyperoxia increased ASK1 phosphorylation with no change in expression of anti- and pro-apoptotic Bcl-2 family proteins (Fig 8B).

## Discussion

Apoptosis of alveolar epithelial cells is considered an important contributor to BPD pathogenesis [47]. Previous work has focused on molecular signals both converging on and being propagated by mitochondria during hyperoxic cell death. Budinger et al. performed a comparative study deleting genes activating intrinsic (Bax, Bak, Bim, Puma, Noxa) and extrinsic (FADD, Bid) apoptotic signaling [48]. Only disruption of *Bax* and *Bak* improved survival of adult mice and mouse embryonic fibroblasts subjected to 95% oxygen. Surprisingly, single deletion of BH3-only (Bim, Puma, and Noxa) direct activators of Bax and Bak did not have any effect. It is possible that BH3-only pathways resulting in activation of Bax and Bak during hyperoxia are redundant and/or converge to repress anti-apoptotic Bcl-2 family proteins such as Bcl-X_L_, Mcl-1, and Bcl-2 [49]. Since intrinsic apoptotic signaling via Bcl-2 family proteins is an important determinant of hyperoxic cell death, we hypothesized that mitochondrial oxidoreductases may serve as a signaling intermediate between intracellular oxidants generated during hyperoxia and injury/death pathways. Our data provide a unique contribution considering how Trx2 and Prx3 function to facilitate electron flux for detoxification of mitochondrial oxidants and regulation of injury signaling during hyperoxic exposure.

In agreement with previous reports, we demonstrate that mtROS directly contribute to oxygen-induced cell death [9, 10]. Continuous MitoTEMPO treatment was able to detoxify mitochondrial superoxide radical generation which correlated with significantly reduced hyperoxic cell death. Superoxide radical was detected with MitoSOX via flow cytometry, which increased emitted red fluorescence by generation of the two-electron oxidation product, 2-hydroxymitoethidium, formed from MitoSOX interactions with superoxide radical [50]. MitoSOX carries a net positive charge and preferentially reacts with superoxide to form 2-hydroxymitoethidium; however, it is possible that a minor quantity of MitoSOX is oxidized by other routes. Reactions with single electron oxidants, such as hydrogen peroxide and peroxidases, result in non-specific oxidation of MitoSOX and produce mitohydroethidium, which also generates red fluorescence [51]. The fluorescent spectra of 2-hydroxymitoethidium, the superoxide specific product, and mitohydroethidium overlap. For this reason, in the context of this study, MitoSOX does not provide a fully direct measure of superoxide production and activity. Rather, it is a sensor of mitochondrial oxidants and suggests that hyperoxia augments generation of mitochondrial oxidants and/or impairs their detoxification. While recognized as an indirect sensor, due to the superoxide specificity of MitoTEMPO [52], these data support that MitoSOX reactivity was, in large part, sensitive to hyperoxic generation of mitochondrial superoxide.

Although mitochondrial hyperpolarization and mtROS generation are associated with MPTP activation [45], MPTP activation may not be required for hyperoxia-induced cell death. Currently, cyclophilin D is the only defined regulatory component of the MPTP and mice deficient in *cyclophilin D* had similar TUNEL-staining, lung pathology, and survival rates as wild-type counterparts during hyperoxic injury [48]. ShRNA knockdown of either Trx2 or Prx3 resulted in increased mtROS and mitochondrial hyperpolarization during culture in both room air and hyperoxia. It is unknown if or how mtROS and mitochondrial hyperpolarization are related during hyperoxic injury but both are likely influenced by bioenergetic changes. For example, less ATP synthesized by OXPHOS during hyperoxic exposure may promote mitochondrial hyperpolarization [53, 54].

Trx2 activity, and not Prx3, was required for cytoprotection from hyperoxic cell death. Reduced Trx2 binds to the N-terminal of ASK1, preventing serine/threonine kinase activity [26]. Our data demonstrate that ASK1 phosphorylation increased during hyperoxic culture and was exacerbated by Trx2 knockdown. As a MAP kinase kinase kinase, ASK1 signaling culminates in JNK and p38 activation, both of which are associated with hyperoxic injury [55–57]. Furthermore, *ASK1*-deficient mice had reduced lung injury when treated with large tidal mechanical ventilation and hyperoxia [58]. Inhibitory Trx2 binding to ASK1 can be relieved by overt thioredoxin oxidation or displacement via TXNIP binding [28, 59]. Although pulmonary TXNIP may inhibit alveolar growth [60], we did not detect mitochondrial localization of TXNIP in A549 cells during hyperoxia (data not shown). ASK1 phosphorylation can also be stimulated by TRAF2 and TRAF6 binding [61]. However, oxidative stimulation may also be required to promote TRAF2 and TRAF6 binding, ASK1 dimerization, and autophosphorylation at Thr845. Trx2 cytoprotection may also involve regulation of Bcl-2 family proteins. Knockdown of Trx2 in DT40 cells caused loss of anti-apoptotic Bcl-X_L_ but this was not dependent on oxidoreductase activity [62]. Modulation of Bcl-X_L_ levels is associated with injury outcomes in hyperoxia [13–16], but we did not detect Trx2-dependent changes in Bcl-X_L_ expression (Fig 8B). It is possible that ASK1 modulation may serve as a mechanistic link between Trx2 and the Bcl-2 family proteins since Bcl-2 is phosphorylated by the ASK1:JNK MAPD signaling axis [63]. This leaves the possibility that post-translational modifications or localization changes in Bcl-2 family proteins is critical for Trx2-mediated cytoprotection from hyperoxic cell death.

**Fig 9:**
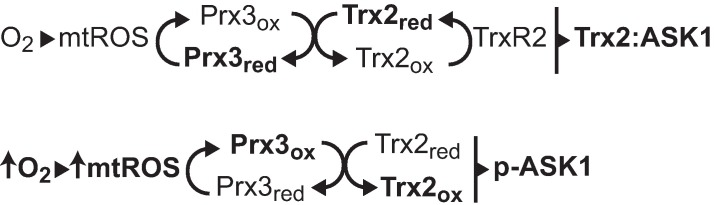
Electron flux via Prx3 and Trx2 during hyperoxic injury. Schematic demonstrating mitochondrial electron flux via the Prx3 and Trx2 under control and hyperoxic conditions.

Independent of precise apoptotic mechanisms, our data support that Prx3 acts as an oxidant sensor to modulator Trx2 activity. The reactivity of MitoSOX with superoxide radical is likely short-lived as it is dismutated to hydrogen peroxide by SOD2. Hydrogen peroxide can initiate redox-dependent signaling changes through reversible oxidation of reactive protein thiols [64]. Although cysteine oxidation by hydrogen peroxide is thermodynamically favorable, direct oxidation of protein thiols is unlikely to occur as the kinetic rate is very slow (*k* ~1–10 M^-1^s^-1^) with the exemption of catalytic thiols of dedicated redox enzymes including catalase, peroxiredoxins, glutathione peroxidases, and ascorbate peroxidases [17, 41, 65]. Prx3 reacts with approximately 90% of mitochondrial peroxides due to abundance and kinetic activity [17]. In contrast to the high buffering capacity of Prx3, Trx2 is present at much lower concentrations and is therefore a limiting factor more likely to serve as a threshold to initiate redox-dependent signaling. As shown in Fig 9, under control conditions, reduction of molecular oxygen by OXPHOS results in formation of mtROS which may be comprised of superoxide radical, hydrogen peroxide, and/or hydroxyl radical. Prx3 reduces mitochondrial peroxide and is recycled by Trx2 and TrxR2 with electrons being ultimately donated from NADPH. Therefore, Trx2 is primarily in a reduced state which exhibits selective inhibition of ASK1. During hyperoxic exposure, accumulation of mtROS and oxidation of Prx3 increases Trx2:TrxR2 redox cycling which likely exhausts reducing equivalents of NADPH [41]. Hyperoxic depletion of NADPH may also explain why overexpression of Trx2 did not reduce oxygen-induced cell death (S5 Fig). Oxidation of Trx2 relieves ASK1 inhibition, resulting in subsequent ASK1 phosphorylation and activation of cell death. It is also possible that other Trx2 substrates serve as mediators between Trx2 oxidation and ASK1 phosphorylation. Since oxidized Prx3 is required to relieve Trx2 anti-apoptotic repression, this scheme describes why limiting either Prx3 or Trx2 results in accumulation of mitochondrial peroxide while inhibition of only Trx2 promotes hyperoxic cell death. Based on this mechanism, we propose that hyperoxic upregulation of Trx2 and TrxR2 is an adaptive response to prevent accumulation of oxidized Prx3 as the initiating damage signal. Although Wnt may influence TrxR2 expression [66], no regulatory elements in the *Trx2* promoter have been described and this is an important trajectory of future research.

Since Prx3 and Trx2 do not exclusively metabolize mitochondrial peroxides, it is important to consider how mitochondrial glutathione peroxidases (Gpx) function in response to hyperoxic injury. It is likely that peroxide metabolism and protein glutathionylation function in parallel in the mitochondria since depletion of glutathione results in Trx2 oxidation [67]. Loss of *Gpx1* did not increase mortality in adult mice treated with hyperoxia, further supporting a redundant functional link between thioredoxin- and glutathione-dependent systems [68]. However, *Trx2*-deficient mice are not viable, marked by apoptosis beginning at E9.5, a time coinciding with a metabolic shift to OXPHOS and likely increase in mtROS generation [69].

Exposure of newborn mice to short-term hyperoxia followed by recovery in room air recapitulates many BPD pathologies including impaired alveolar and vascular growth with restrictive lung disease and early mortality attributed to pulmonary hypertension [5, 70–72]. A possible explanation for these primary and subsequent phenotypes is that neonatal hyperoxia reduces numbers of alveolar epithelial AT2 cells [4–6], which are known to serve as progenitor cells during alveologenesis and alveolar repair [73]. Although it is possible that Trx2 mediates AT2 cell death *in vivo*, it is unlikely that hyperoxic depletion of AT2 cells in the perinatal lung can be explained exclusively by cell death. Yee et al. report that hyperoxia stimulates expression of genetic markers indicative of alveologenesis and that AT2 cell numbers slowly diminish during the recovery phase [6]. This exacerbated expansion of AT2 cells may be due to prolonged or enhanced activation of oxygen-dependent signaling pathways regulating perinatal alveologenesis while transitioning to a more oxidative atmospheric environment outside the womb. Therefore, altered AT2 programming may also contribute to BPD pathologies due to accumulation of mitochondrial oxidants in the alveolar epithelium due to tissue hypoxia, hyperoxic exposure, and recovery to room air. While hyperoxic injury has direct translational implications in BPD, production of mtROS is an important component of oxidative lung diseases including acute lung injury (ALI) and acute respiratory distress syndrome (ARDS) [74, 75]. It is likely that Trx2 and Prx3 are similarly involved in maintenance of mtROS and apoptotic signaling during ALI and ARDS which may mediate inflammatory signaling via mitochondrial release of damage-associated molecular patterns (DAMPs) [76].

Taken together, our data support that Trx2 activity is cytoprotective against hyperoxic injury; however, these results obtained from adenocarcinoma cells may not accurately recapitulate molecular physiologies *in vivo* due to redox and metabolic imbalances in cell culture models [77, 78], and altered expression of Prx3, Trx2, or TrxR2 which will influence electron flux [79]. Consistent with such differences, no changes in expression of Prx3, Trx2, or TrxR2 were detected in lungs of oxygen-exposed newborn mice (S6 Fig). Despite these preliminary findings, we still speculate that future studies should consider accumulation of mitochondrial peroxides and altered redox signaling as molecular mechanisms involved in AT2 programming during lung development and oxidative lung injuries. In summary, these data support that detoxification of mitochondrial oxidants was uncoupled from cytoprotection which we hypothesize occurs through an ASK1 thiol switch regulated by Trx2 oxidation. Oxidized Prx3 accumulates during hyperoxic exposure and serve as the initiating event by exhausting redox cycling of Trx2/TrxR2, thus relieving Trx2-dependent inhibition of ASK1 phosphorylation. Prx3, Trx2, and TrxR2 function as a system to detoxify mitochondrial peroxides during hyperoxic injury although it is likely that their activities are coordinated with catalase, manganese SOD, and Gpx1. Investigation of coordination between these pathways may reconcile conflicting data from transgenic and knockout antioxidant enzyme mouse models [80, 81].

## Conclusions

We conclude that Prx3 and Trx2 comprise an adaptive system to sense changes in atmospheric oxygen tension and influence cellular injury responses through both detoxification of mitochondrial oxidants and regulation of mitochondrial redox-dependent signaling.

## Supporting Information

S1 Fig Flow cytometric analysis of hyperoxic cell death. A549 cells were cultured in room air or hyperoxia for four days. Viability was measured via flow cytometry by using TOPRO-3 as a marker of cell death (percentages indicate upper-quadrant distribution of cells).
(EPS)

S2 Fig Protein quantification of mitochondrial redoxins Prx3, Trx2, and TrxR2 during hyperoxia. Densitometric quantification of SDS-PAGE/immunoblots of oxygen-treated A549 and H1299 cell lysates probed for (A) Prx3, (B) Trx2, and (C) TrxR2 normalized to β-actin as a loading. Data are expressed as mean ± standard deviation of 3 biological replicates analyzed by one-way ANOVA. Statistical significance was defined as *p<0.05, **p<0.01, and †p<0.001.
(EPS)

S3 Fig Decreased mitochondrial mass following ddC or EtBr treatment. Ratio of mitochondrial:nuclear Ct values for *D-Loop* and *COX1* after normalization to *β2M* quantified by qPCR in A549 cells following 5 days treatment with 20 μM dideoxycytidine (ddC) or 75 ng/mL ethidium bromide (EtBr). Data are expressed as mean ± standard deviation of 3 biological replicates analyzed by one-way ANOVA. Statistical significance was defined as **p<0.01 and †p<0.001.
(EPS)

S4 Fig Detection of Trx2 and Prx3 oxidation. Differential (A) Trx2 and (B) Prx3 thiol labeling by AMS and NEM respectively.
(EPS)

S5 Fig Trx2 overexpression does not prevent hyperoxic cell death. A C-terminal flag epitope was introduced by PCR of *Trx2* cDNA (NM_012473), ligated into the doxycycline-inducible pBIG2i vector, and stably transfected into H1299 cells. (A) SDS-PAGE/immunoblot of H1299 cell lysates for the Trx2-flag transgene 24 hours following culture in 2μg/mL doxycycline (DOX). (B) Immunocytochemistry and mitochondrial co-localization of Trx2-flag. (C) Viability of two H1299+Trx2-flag clones cultured in absence or presence of DOX and cultured in hyperoxia for 3 days. Data are expressed as mean ± standard deviation and analyzed by one-way ANOVA.
(EPS)

S6 Fig Oxygen-dependent pulmonary expression of Prx3, Trx2, and TrxR2. C57Bl/6J newborn litters (PND<0.5) were randomly placed in 85% oxygen or room air for the first seven days of life. After seven days, room air or hyperoxic lungs were analyzed by qPCR using the following Taqman probes: Txn2 (MM0044931_M1), Prdx3 (MM00545848_M1), Txnrd2 (MM00496766_M1), and HPRT (MM01545399_M1). Data are expressed as mean ± standard deviation of 3–4 biological replicates analyzed by student’s t-test.
(EPS)

S1 Table qPCR primer and probe sequences. qPCR primers and 6-carboxyfluorescein (FAM)-labeled probe sequences targeting human Txn2, Prx2, TrxR2, and GAPDH.
(DOCX)

S2 Table qPCR primer and probe sequences for quantifying mitochondrial mass. Catalog numbers and sequence for qPCR primers and 4,7,2-tricholo-7-phenyl-carboxyfluorescein (VIC)-labeled probe sequences targeting human D-Loop, COX1, and β-2-microglobulin.
(DOCX)

S3 Table ShRNA sequences. Non-targeting or human Trx2- and Prx3-targeting shRNA sequences (sense-loop-antisense).
(DOCX)
