# Supplementary material for: Detoxification of Mitochondrial Oxidants and Apoptotic Signaling Are Facilitated by Thioredoxin-2 and Peroxiredoxin-3 during Hyperoxic Injury
Source: PLoS One. 2017 Jan 3;12(1):e0168777. doi: 10.1371/journal.pone.0168777 (PMC5207683; doi:10.1371/journal.pone.0168777)
Supplement: S1 Table — qPCR primers and 6-carboxyfluorescein (FAM)-labeled probe sequences targeting human Txn2, Prx2, TrxR2, and GAPDH. (DOCX) [file pone.0168777.s007.docx]

**S1 Table. qPCR primer and probe sequences.**

qPCR primers and 6-carboxyfluorescein (FAM)-labeled probe sequences targeting human Txn2, Prx2, TrxR2, and GAPDH.

| **Target** | **mRNA primer sequence 5’→3’** | **Accession Number** |
| --- | --- | --- |
| Trx2 | Fwd: GCCAAGGTGGATATTGATG | nm003329.3 |
|  | Rev: ACGTCCCCATTCTTCATG |  |
|  | Probe: [FAM]CACCGCTGACACCTCATACTCA |  |
|  |  |  |
| Prx3 | Fwd:CCAGTCAAGCAAAATTATTC | [nm001302272.1](http://www.ncbi.nlm.nih.gov/nuccore/NM_001302272.1) |
|  | Rev:GGCTGTACCCTTAAAATAG |  |
|  | Probe: [FAM]CACCAGTTCCTCATGCCATGC |  |
|  |  |  |
| TrxR2 | Fwd: GAGGCCTGATCCAAGATG | nm003330.2 |
|  | Rev: CGTGATTTTGAACAGCTTC |  |
|  | Probe: [FAM]TCTTCCTCCAGTCATGCGGC |  |
|  |  |  |
| GAPDH | Fwd: CATCCATGACAACTTTGGTA | nm002046.4 |
|  | Rev: CCATCCACAGTCTTCTGG |  |
|  | Probe: [FAM]ACCACAGTCCATGCCATCACT |  |
