# Supplementary material for: Detoxification of Mitochondrial Oxidants and Apoptotic Signaling Are Facilitated by Thioredoxin-2 and Peroxiredoxin-3 during Hyperoxic Injury
Source: PLoS One. 2017 Jan 3;12(1):e0168777. doi: 10.1371/journal.pone.0168777 (PMC5207683; doi:10.1371/journal.pone.0168777)
Supplement: S2 Table — Catalog numbers and sequence for qPCR primers and 4,7,2-tricholo-7-phenyl-carboxyfluorescein (VIC)-labeled probe sequences targeting human D-Loop, COX1, and β-2-microglobulin. (DOCX) [file pone.0168777.s008.docx]

**S2 Table. qPCR primer and probe sequences for quantifying mitochondrial mass.**

Catalog numbers and sequence for qPCR primers and 4,7,2-tricholo-7-phenyl-carboxyfluorescein (VIC)-labeled probe sequences targeting human D-Loop, COX1, and β-2-microglobulin.

|  | | |
| --- | --- | --- |
| **Target** | **primer sequence 5’→3’** | **Thermo Fisher Assay Number** |
| D-Loop (MT-7S) |  | Hs02596861_s1 |
|  |  |  |
| COX1 (MT-CO1) |  | Hs02596864_g1 |
|  |  |  |
| β-2-microglobulin |  |  |
| FWD | GCTGGGTAGCTCTAAACAATGTATTCA |  |
| REV | CCATGTACTAACAAATGTCTAAAATGGT |  |
| PROBE | [VIC]CAGCAGCCTATTCTGC[VIC] |  |
|  |  |  |
