# Supplementary material for: Detoxification of Mitochondrial Oxidants and Apoptotic Signaling Are Facilitated by Thioredoxin-2 and Peroxiredoxin-3 during Hyperoxic Injury
Source: PLoS One. 2017 Jan 3;12(1):e0168777. doi: 10.1371/journal.pone.0168777 (PMC5207683; doi:10.1371/journal.pone.0168777)
Supplement: S3 Table — Non-targeting or human Trx2- and Prx3-targeting shRNA sequences (sense-loop-antisense). (DOCX) [file pone.0168777.s009.docx]

**S3 Table. ShRNA sequences.**

Non-targeting or human Trx2- and Prx3-targeting shRNA sequences (sense-loop-antisense).

|  | |
| --- | --- |
| **Target** | **Sequence 5’→3’ (sense-loop-antisense)** |
| Non-targeting | ctaccgttgttataggtgttcaagagacacctataacaacggtag |
|  |  |
| Trx2 | GGATCTCCTTGACAACCTTTATTCAAGAGATAAAGGTTGTCAAGGAGATCC |
|  |  |
| Prx3 | GCACTCTTGTCAGACTTAACTTTCAAGAGAAGTTAAGTCTGACAAGAGTGC |
